# Defective Expression of Mitochondrial, Vacuolar H^+^-ATPase and Histone Genes in a *C. elegans* Model of SMA

**DOI:** 10.3389/fgene.2019.00410

**Published:** 2019-05-03

**Authors:** Xiaoyang Gao, Jing Xu, Hao Chen, Dingwu Xue, Wenju Pan, Chuanman Zhou, Yongchao C. Ma, Long Ma

**Affiliations:** ^1^Center for Medical Genetics, School of Life Sciences, Central South University, Changsha, China; ^2^Department of Pediatrics, Northwestern University Feinberg School of Medicine, Ann & Robert H. Lurie Children’s Hospital of Chicago, Chicago, IL, United States; ^3^Hunan Key Laboratory of Medical Genetics, School of Life Sciences, Central South University, Changsha, China; ^4^Hunan Key Laboratory of Animal Models for Human Diseases, School of Life Sciences, Central South University, Changsha, China

**Keywords:** SMN, mitochondria, vacuolar H^+^-ATPase, histone, *C. elegans*

## Abstract

Spinal muscular atrophy (SMA) is a severe motor neuron degenerative disease caused by loss-of-function mutations in the survival motor neuron gene *SMN1*. It is widely posited that defective gene expression underlies SMA. However, the identities of these affected genes remain to be elucidated. By analyzing the transcriptome of a *Caenorhabditis elegans* SMA model at the pre-symptomatic stage, we found that the expression of numerous nuclear encoded mitochondrial genes and vacuolar H^+^-ATPase genes was significantly down-regulated, while that of histone genes was significantly up-regulated. We previously showed that the *uaf-1* gene, encoding key splicing factor U2AF large subunit, could affect the behavior and lifespan of *smn-1* mutants. Here, we found that *smn-1* and *uaf-1* interact to affect the recognition of 3′ and 5′ splice sites in a gene-specific manner. Altogether, our results suggest a functional interaction between *smn-1* and *uaf-1* in affecting RNA splicing and a potential effect of *smn-1* on the expression of mitochondrial and histone genes.

## Introduction

Spinal muscular atrophy (SMA) is a severe congenital motor neuron degenerative disease caused by loss-of-function mutations in the survival motor neuron gene 1 (*SMN1*) (Lefebvre et al., 1995). In addition to *SMN1*, humans also carry *SMN2*, which is different from *SMN1* by a single nucleotide, leading to defective RNA splicing and reduced full length functional SMN protein expression (Kashima and Manley, 2003; Cartegni et al., 2006). *SMN* is conserved and only one *SMN* gene is found in mouse, *Drosophila* and *Caenorhabditis elegans* (Edens et al., 2015).

A key function of SMN is to facilitate the proper assembly of the small nuclear ribonucleoproteins (snRNP) (Fischer et al., 1997; Liu et al., 1997; Pellizzoni et al., 1998). snRNPs form the core components of the pre-mRNA splicing machinery (Reed, 2000; Makarov et al., 2002). It is hypothesized that defective snRNP formation caused by *SMN* deficiency and subsequent aberrant gene splicing underlie the pathogenesis of SMA (Burghes and Beattie, 2009). However, the key molecular changes caused by *SMN* deficiency that lead to SMA and *SMN* mutant phenotype remain to be elucidated.

In addition to snRNP biogenesis and pre-mRNA splicing, SMN also affects mRNA localization in neurons (Rossoll et al., 2003), mRNA local translation (Kye et al., 2014), axonal guidance (McWhorter et al., 2003), muscle functions (Rajendra et al., 2007), endocytosis (Dimitriadi et al., 2016; Hosseinibarkooie et al., 2016; Riessland et al., 2017), autophagy (Garcera et al., 2013; Custer and Androphy, 2014) and multiple other RNA-related processes (Singh et al., 2017). *SMN* may interact with numerous genes in *Drosophila* and *C. elegans* (Chang et al., 2008; Dimitriadi et al., 2010; Sen et al., 2013). The broad function of *SMN* implicates that SMA pathogenesis might involve molecular processes beyond defective RNA splicing and gene expression.

The nematode *C. elegans* carries an ortholog of *SMN* named *smn-1* and has been used for studying *in vivo* functions of *SMN* (Bertrandy et al., 1999; Miguel-Aliaga et al., 1999; Burt et al., 2006; Briese et al., 2009; Dimitriadi et al., 2010, 2013, 2016; Sleigh et al., 2011; Gao et al., 2014). *smn-1* is essential for *C. elegans* survival (Miguel-Aliaga et al., 1999). A deletion mutation of *smn-1*, *ok355*, causes developmental arrest, a reduced lifespan and a progressive loss of motor functions that can be partially attributed to neuronal defects (Briese et al., 2009; Dimitriadi et al., 2010).

One approach to understand SMA is to investigate how SMN interacts with other splicing factors to affect gene expression and RNA splicing. In eukaryotes, pre-mRNA splicing (RNA splicing) begins with the recognition of 5′ splice sites by the U1 snRNP and of 3′ splice sites by the U2AF large and small subunits (Reed, 2000). Specifically, the U2AF large subunit (UAF-1 in *C. elegans*) and small subunit (UAF-2) interact with each other to bind the pyrimidine tract preceding the 3′ splice site and the conserved 3′ splice site (Reed, 2000; Maniatis and Reed, 2002).

A complete loss of function in UAF-1 or UAF-2 leads to lethality in *C. elegans* (Zorio and Blumenthal, 1999; Ma and Horvitz, 2009). We previously isolated a missense mutation in *uaf-1* (Thr180Ile in *C. elegans*, Thr145Ile in mouse) that causes conditional lethality at higher temperatures and affects alternative splicing (Ma and Horvitz, 2009; Ma et al., 2011). An exploration of how *uaf-1* interacts with *smn-1* found that this *uaf-1* mutation (*n4588*) and a derived *uaf-1* mutation (*n4588 n5127*, T180I and M157I) (Ma and Horvitz, 2009) can partially suppress the behavioral and lifespan defects of *smn-1(ok355)* mutants at late developmental stages when *smn-1* mutants exhibit severe defects in behavior and lifespan (Gao et al., 2014). Since *uaf-1(n4588)* can decrease or increase the recognition of 3′ splice sites in a gene-specific manner (Ma and Horvitz, 2009; Ma et al., 2011; Zhou et al., 2018), we postulate that this mutation might improve the splicing of a subset of affected genes in *smn-1(ok355)* mutants, which might compensate the defects in snRNPs and lead to the suppression (Gao et al., 2014). In this study, we examined how *smn-1* affected *C. elegans* gene expression and RNA splicing by analyzing the transcriptome of *smn-1* mutants at the pre-symptomatic stage.

## Results

### *smn-1(ok355)* Affects the Expression of a Wide Array of Genes in *C. elegans*

It was unclear how *smn-1* affects gene expression in *C. elegans*. *ok355* is a null mutation in *smn-1* that deletes the majority of the coding regions of the gene (Briese et al., 2009). *smn-1(ok355)* mutants have similar behavior, body size and survival rate at early larval stages compared to wild type (Briese et al., 2009). The mutants start to exhibit gradual deterioration in locomotion and pharyngeal pumping after 2 days post the L1 stage (Briese et al., 2009; Gao et al., 2014), probably due to the depletion of maternally inherited *smn-1* products. We therefore used animals at this stage (2 days after L1) for RNA-Seq analyses, postulating that we might observe early-stage changes of gene expression caused by *smn-1* deficiency.

**FIGURE 1:**
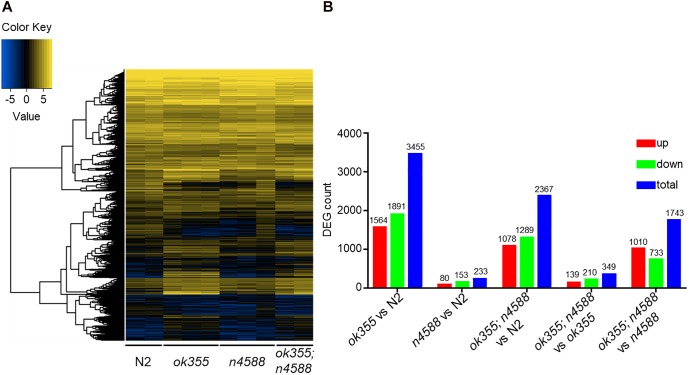
RNA-Seq identified differentially expressed genes (DEGs) in *smn-1(ok355)* mutants. **(A)** Heat map of DEGs in mutants and wild type. Blue color indicates low expression and yellow color indicates high expression. **(B)** Numbers of DEGs in mutants compared to wild type or in *smn-1(ok355); uaf-1(n4588)* double mutants compared to *smn-1(ok355)* or *uaf-1(n4588)* single mutants.

We performed RNA-Seq on animals of different genotypes and focused on differentially expressed genes (DEGs) with *q* ≤ 0.05 (adjusted *p*-value) and | log_2__ratio|≥ 1 from pairwise comparisons. The heat map indicates that *smn-1(ok355)* mutants exhibited apparently different gene expression profiles compared to wild type (Figure 1A). Three thousand four hundred and fifty-five DEGs were identified in *smn-1(ok355)* mutants, including 1564 up-regulated and 1891 down-regulated ones (Figure 1B, *ok355* vs. N2 and Supplementary Table S1). It is interesting to note that the expression levels of numerous genes were altered significantly beyond two folds. This is exemplified by the fold changes of the top 200 up-regulated and the top 200 down-regulated DEGs (Supplementary Table S2), among which *smn-1* is the third most down-regulated one. Consistently, the raw sequencing reads and FPKMs (fragments per kilobase of transcript per million mapped reads) of *smn-1* transcripts were barely detectable in all *smn-1(ok355)* samples compared to wild type or *uaf-1(n4588)* samples (Supplementary Table S3).

### *smn-1(ok355)* Mutants Have Defective Mitochondrial, Vacuolar H^+^-ATPase and Histone Gene Expression

**FIGURE 2:**
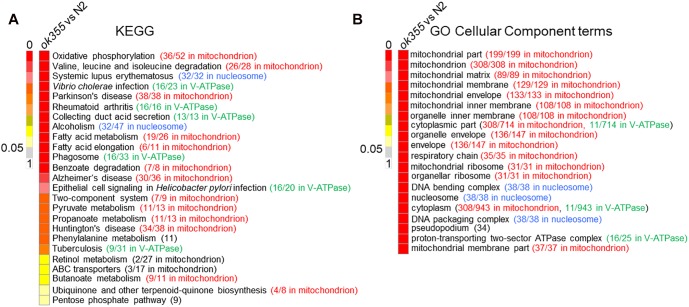
Kyoto Encyclopedia of Genes and Genomes (KEGG) pathway analysis and gene ontology (GO) cellular component analysis in *smn-1(ok355)* single mutants. **(A)** Significantly affected KEGG pathways in *smn-1(ok355)* mutants. The number of DEGs with similar annotated functions vs. total DEGs in each pathway is shown in parentheses. Mitochondrial genes are indicated in red, V-ATPase genes in green and histone genes in blue. **(B)** Top 20 significantly affected GO cellular component terms in *smn-1(ok355)* mutants. Mitochondrial genes are indicated in red, V-ATPase genes in green and histone genes in blue. The number of DEGs in each term is shown in the parentheses. The colors of the scale bars indicate the statistical significance (*q*-value) of the affected pathways or terms, with *q*-value above 0.05 considered non-significant.

To identify the major biological pathways affected by *smn-1*, we performed KEGG (Kyoto Encyclopedia of Genes and Genomes^1^) pathway analyses on the DEGs of *smn-1(ok355)* mutants. Twenty-five significantly affected pathways were identified (Figure 2A and Supplementary Table S4), 13 of which were enriched with mitochondrial genes (Figure 2A, highlighted in red), followed by six pathways enriched with genes encoding vacuolar H^+^-ATPases (V-ATPases, green) and two pathways enriched with genes encoding nucleosome proteins (histones, blue) (Figure 2A).

The other four KEGG pathways significantly affected in *smn-1(ok355)* mutants include phenylalanine metabolism, retinol metabolism, ATP-binding cassette (ABC) transporters and the pentose phosphate pathway (Figure 2A). Two DEGs in the phenylalanine metabolism pathway were *bas-1* and *tdc-1*, which are required for the synthesis of neurotransmitters serotonin (*bas-1*) (Hare and Loer, 2004), tyramine (*tdc-1*) and octopamine (*tdc-1*) (Alkema et al., 2005) (Supplementary Table S4). Among the 27 DEGs in the retinol metabolism pathway, 12 were *ugt* genes encoding UDP glycosyltransferases (UGT), followed by seven *cyp* genes encoding cytochrome P450 proteins (Supplementary Table S4). UGT and P450 interact and play essential roles in xenobiotic and endobiotic metabolism (Ishii et al., 2010; Knights et al., 2013). Among the 17 DEGs encoding ABC transporters, three were similar to yeast homologs localized in mitochondria (Figure 2A and Supplementary Table S4, based on annotations^2^), while the others were similar to homologs localized on the plasma membrane or other organelles (Supplementary Table S4). The pentose phosphate pathway is an important route for generating NADPH and maintaining redox balance (Stincone et al., 2015), a function also shared by mitochondria.

Considering that the same gene can be assigned to different KEGG pathways, we further sorted these DEGs to a non-redundant list (Supplementary Table S5). In the list, the top groups include genes that encode mitochondrial proteins (94 genes), histones (32), ABC transporters (17), V-ATPases (16), UGTs (12), phosphoprotein phosphatases (12), C-type lectins (10), and cytochrome P450 (7) (Supplementary Table S5).

Gene ontology (GO^3^) analyses of the DEGs showed that mitochondrial components were the most significantly affected group (Figure 2B, red), similar to the KEGG analyses. Components of the nucleosome proteins and V-ATPases were also among the top 20 categories (Figure 2B, blue and green, respectively). Non-redundant DEGs related to these GO terms are listed in Supplementary Table S6.

Though no KEGG pathways or GO terms related to lifespan or motor functions were predicted to be significantly affected in *smn-1(ok355)* mutants based on the hypergeometric test (Materials and Methods), a closer examination of the RNA-Seq results identified a non-redundant list (189 in total) of DEGs annotated to GO terms related to lifespan, motor functions and/or neuron activities (Supplementary Table S7). In the list, 15 were mitochondrial genes (Supplementary Table S7, highlighted in red) and two were V-ATPase genes (Supplementary Table S7, highlighted in green).

### Validation of Down-Regulated Gene Expression in *smn-1(ok355)* Mutants

**FIGURE 3:**
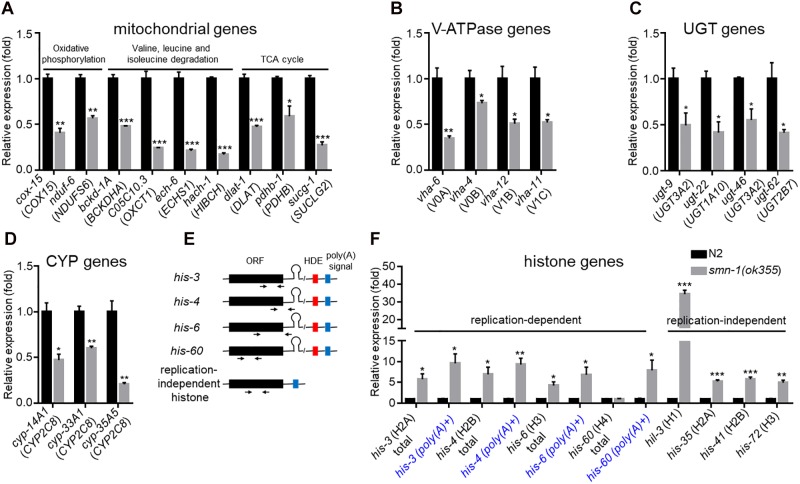
RT-qPCR validation of DEGs in *smn-1(ok355)* single mutants. Relative expression of mitochondrial genes **(A)**, V-ATPase subunit genes **(B)**, *ugt* genes **(C)**, and *cyp* genes **(D)** in *smn-1(ok355)* mutants. The corresponding mammalian homologs are indicated in parentheses. **(E)** Gene structures of replication-dependent and -independent histone genes and positions of PCR primers. HDE (red): presumably *C. elegans*-specific histone downstream element (Keall et al., 2007). *Poly(A)* signals are indicated as blue boxes. **(F)** Validation of histone gene expression. For replication-dependent histone genes, total transcripts and *poly(A)+* transcripts were reversely transcribed using random primers and oligo(dT) primers, respectively. Each dataset is the average of three biological replicates. Statistics: two-tailed unpaired Student’s *t*-test. ^∗^*p* < 0.05; ^∗∗^*p* < 0.01; ^∗∗∗^*p* < 0.001. Error bars: standard errors.

To validate the RNA-Seq results, we used RT-qPCR to quantify the expression of a group of down-regulated DEGs randomly selected in the mitochondrial, V-ATPase, *ugt* and *cyp* groups (Supplementary Table S8, highlighted in red). Among the 13 nuclear genome-encoded mitochondrial genes examined (Supplementary Table S8), nine were confirmed to be down-regulated to ∼50% or less of the wild-type levels (Figure 3A), while four were not (Supplementary Figure S1). However, the differential expression of the four mitochondrial genome-encoded genes was not validated (Supplementary Figure S1). We examined seven V-ATPase genes (Supplementary Table S8) and validated that four were down-regulated (Figure 3B and Supplementary Figure S1). We further validated the down-regulation of four *ugt* genes (Figure 3C and Supplementary Figure S1) out of five examined (Supplementary Table S8) and of three *cyp* genes (Figure 3D and Supplementary Figure S1) out of five examined (Supplementary Table S8). Together, the total validation rate was 20/34.

### Validation of Histone Gene Expression

Different from that of mitochondrial and V-ATPase genes, the expression of the majority of histone genes (34/38, excluding *his-72*, see below) was found to be significantly up-regulated in *smn-1(ok355)* mutants based on RNA-Seq results (Supplementary Table S8).

Histone genes can be classified into two types: the replication-dependent type, which produces mature mRNA transcripts with a unique 3′ stem-loop end structure without *poly(A)* tails [*poly(A)-*], and the replication-independent type, which produces transcripts with *poly(A)* tails [*poly(A)+*] (Marzluff et al., 2008) (Figure 3E). For replication-dependent histone genes, studies in mammalian *SMN*-deficient cells showed that defective cleavage between the 3′ stem-loop end structure and the *poly(A)* signal (Figure 3E) by the U7 snRNP would lead to the accumulation of *poly(A)+* transcripts (Tisdale et al., 2013).

To validate histone gene expression, we selected four genes from both replication-dependent and -independent types, each of which encodes a different histone (Supplementary Table S8 and Figure 3F). It is worth noting that *his-72*, which encodes an H3 histone and is replication-independent, was not identified as a DEG based on RNA-Seq (Supplementary Table S8). We included it as a representative of its type (replication-independent H3-encoding).

For replication-independent histone genes, we generated cDNAs using random oligo primers in reverse transcription experiments (see section “Materials and Methods”). RT-qPCR experiments confirmed that the expression levels of the four selected genes (Supplementary Table S8) were significantly up-regulated in *smn-1(ok355)* mutants (Figure 3F).

The RNA-Seq results suggested up-regulation of *poly(A)+* transcripts of replication-dependent histone genes in *smn-1(ok355)* mutants (Supplementary Table S8). To validate the change, we generated cDNAs for either *poly(A)+* transcripts or total transcripts (see section “Materials and Methods”) of four such genes. RT-qPCR experiments confirmed that the *poly(A)+* transcripts were significantly up-regulated [Figure 3F, e.g., *his-3 (poly(A)+)*].

Interestingly, the total transcript levels of three replication-dependent histone genes were also up-regulated in *smn-1(ok355)* mutants (Figure 3F), except for *his-60*.

**FIGURE 4:**
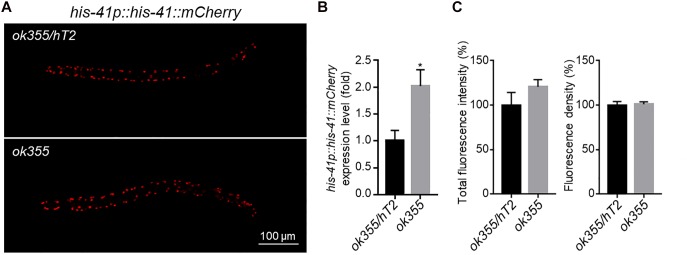
*his-41p::his-41::mCherry* transgene expression. **(A)** Representative fluorescence pictures of HIS-41::mCherry fusion protein expression in an *smn-1(ok355)/hT2* heterozygous or an *smn-1(ok355)* homozygous animal. **(B)** RT-qPCR quantification of *his-41::mCherry* expression levels. **(C)** Quantification of the total fluorescence intensity (left) and density (right) of mCherry. For mRNA expression, each dataset is the average of three biological replicates from one transgenic line. For mCherry intensity, each dataset is the average of 20 transgenic animals in two transgenic lines, with 10 animals from each line. Statistics: two-tailed unpaired Student’s *t*-test. ^∗^*p* < 0.05. Error bars: standard errors.

To investigate whether more histone transcripts would lead to increased protein expression, we generated transgenic animals expressing a *his-41p::his-41::mCherry* transgene (Figure 4A). Consistent with the up-regulation of endogenous histone genes by *smn-1(ok355)* (Figure 3F), the expression of the transgene was increased by ∼100% in *smn-1(ok355)* homozygotes compared to *smn-1(ok355)/+* heterozygotes (Figure 4B). However, the mCherry fluorescence signals were similar between the two genotypes (Figure 4A,C). Similar observations were also made for *his-35* and *hil-3* (XG and LM, unpublished observations). Therefore, a transcript-independent mechanism(s) might be employed to regulate histone protein levels *in vivo*.

### The Effect of *uaf-1(n4588)* on DEGs of *smn-1(ok355)* Mutants

We previously found that the *uaf-1(n4588)* and *uaf-1(n4588 n5127)* mutations can partially suppress the defective behavior and lifespan of *smn-1(ok355)* mutants at late developmental stages when the defects are obvious (Gao et al., 2014). To understand how *uaf-1(n4588)* affects gene expression, we performed RNA-Seq on *uaf-1(n4588)* single and *smn-1(ok355); uaf-1(n4588)* double mutants at the pre-symptomatic stage (Figure 1A).

In *smn-1(ok355); uaf-1(n4588)* double mutants, the major KEGG pathways identified in *smn-1(ok355)* single mutants were no longer significant (Supplementary Figure S2 and Supplementary Tables S4, S9), though many DEGs were shared between the two mutants (Supplementary Tables S5, S6). We postulate that false negatives in the double mutants might render these pathways insignificant. To test this possibility, we compared the expression of eight genes deemed DEGs in *smn-1(ok355)* single mutants but not DEGs in the double mutants (Supplementary Figure S3A). Five of the eight genes had been validated before (Figure 3A,B), while three had not been. The RT-qPCR results showed that seven genes were false negatives in the double mutants (Supplementary Figures S3B,C, *cox-15*, *C05C10.3*, *acdh-2*, *dhs-26*, *vha-6*, *vha-11* and *vha-12*). One gene appeared to be a real negative in the double mutants (Supplementary Figure S3B, *T22B7.7*).

The gene expression profiles of *uaf-1(n4588)* single mutants were similar to that of wild type (Figure 1A). 233 DEGs were identified in *uaf-1(n4588)* mutants, including 80 up-regulated and 153 down-regulated ones (Figure 1B). No KEGG pathways were significantly affected by these DEGs. A few affected GO terms include collagens or structural proteins of the cuticle (Supplementary Table S10). A comparison between *uaf-1(n4588)* single and *smn-1(ok355); uaf-1(n4588)* double mutants identified 1,743 DEGs, among which 1,010 were up-regulated and 733 down-regulated (Figure 1B).

We compared *smn-1(ok355); uaf-1(n4588)* double mutants with *smn-1(ok355)* single mutants and identified 349 DEGs (Figure 1B and Supplementary Table S11). No KEGG pathways were significantly affected by these DEGs. The two GO terms significantly affected were extracellular region and nutrient reservoir activity (Supplementary Table S11). We also identified individual DEGs related to life span, motor functions and neuron activities (Supplementary Table S11).

One hundred and fifty-two of the 349 DEGs in *smn-1(ok355); uaf-1(n4588)* vs. *smn-1(ok355)* comparison exhibited expression changes opposite to that in *smn-1(ok355)* vs. N2 comparison (Supplementary Table S12), among which the expression of 31 down-regulated genes in *smn-1(ok355)* was increased in the double mutants, while that of 121 up-regulated genes in *smn-1(ok355)* was decreased in the double mutants. Though it is interesting to speculate that some of these expression changes might underlie the suppression of *smn-1(ok355)* defects by *uaf-1(n4588)*, caution has to be exercised in interpreting these results before the expression is validated and extensive analyses of gene functions are performed.

### The Effect of *smn-1(ok355)* on Alternative Splicing

Our RNA-Seq results provide a list of candidate DEGs with altered splicing in *smn-1(ok355)* mutants, among which 18 genes (Supplementary Table S13) belonged to the mitochondrial and V-ATPase group. However, the predicted alternative splicing in 17 genes could not be validated by RT-PCR (Supplementary Table S13). The predicted intron retention was validated for the *cpt-2* gene (Supplementary Table S13), while no significant difference was found between wild type and *smn-1(ok355)* mutants (Supplementary Table S13).

**FIGURE 5:**
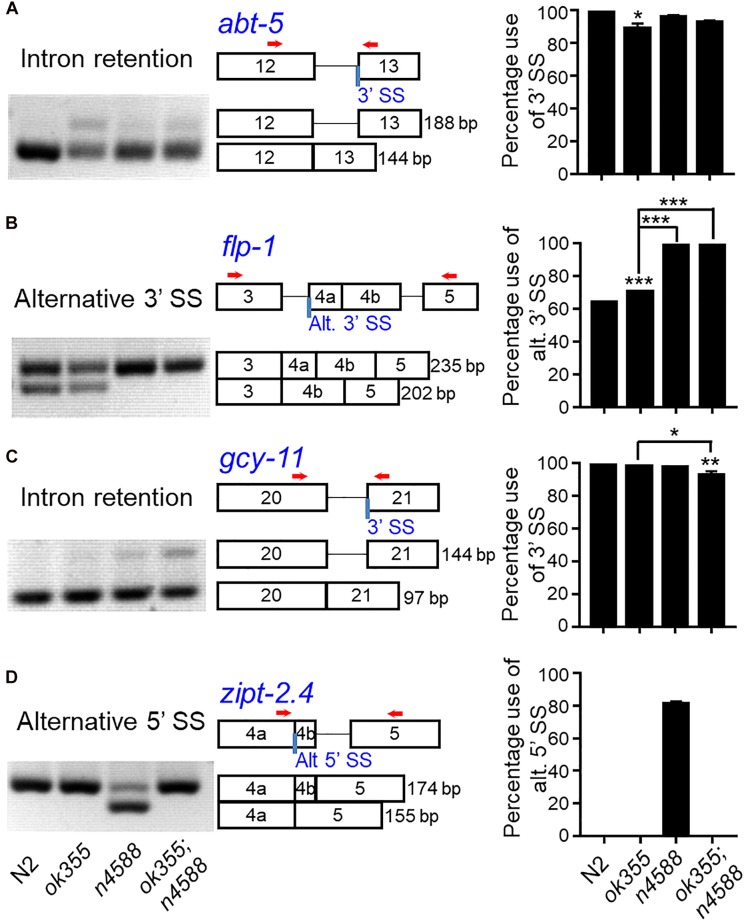
RT-PCR examination of alternative splicing in *smn-1(ok355)* mutants. *smn-1(ok355)* increased the intron retention of the *abt-5* gene **(A)** and the recognition of an alternative 3′ splice site of the *flp-1* gene **(B)**. **(C)** *smn-1(ok355)* and *uaf-1(n4588)* together can increase the intron retention of the *gcy-11* gene. **(D)** *smn-1(ok355)* can strongly suppress the recognition of a cryptic 5′ splice site in *uaf-1(n4588)* mutants. Representative gel pictures, gene structures and splicing quantifications are shown from left to right, with genotypes indicated at the bottom. Red arrows: positions of PCR primers. Each dataset is the average of three biological replicates. Statistics: Bonferroni multiple comparison with one-way ANOVA. ^∗^*p* < 0.05; ^∗∗^*p* < 0.01; ^∗∗∗^*p* < 0.001. Error bars: standard errors.

To further investigate how *smn-1(ok355)* affected alternative splicing, we examined nine genes (Figure 5 and Supplementary Figure S4) that exhibited altered splicing in *uaf-1(n4588)* mutant embryos. These genes were recently reported in a study about the interaction of *uaf-1* with the RNA-binding motif gene *rbm-5* (Zhou et al., 2018).

At the pre-symptomatic larval stage, RT-PCR showed that *smn-1(ok355)* could apparently increase the intron retention of the *abt-5* gene (Figure 5A) and the splicing at an alternative 3′ splice site of the *flp-1* gene (Figure 5B). *smn-1(ok355)* also appeared to act together with *uaf-1(n4588)* to increase the intron retention of the *gcy-11* gene (Figure 5C). Surprisingly, *smn-1(ok355)* completely suppressed the recognition of a cryptic 5′ splice site within the *zipt-2.4* gene in *uaf-1(n4588)* mutants, though *smn-1* had no effect by itself (Figure 5D). For the other five genes, *smn-1(ok355)* did not alter their splicing in either wild-type or *uaf-1(n4588)* background (Supplementary Figure S4).

## Discussion

In this study, we analyzed the global gene expression of a *C. elegans* model of SMA at the pre-symptomatic stage and validated the down-regulation of a subset of genes encoding mitochondrial, vacuolar H^+^-ATPase, UGTs and cytochrome P450. Different from mitochondrial and other genes, most histone genes were up-regulated in this model. Our findings suggest that abnormal expression of mitochondrial, V-ATPase and histone genes could be considered in understanding SMA pathogenesis.

### Defective Expression of Mitochondrial and V-ATPase Genes in *SMN* Mutants

Abnormal expression and/or splicing of numerous genes are found in different SMA models, including human, mouse, zebrafish, *Drosophila*, *C. elegans* and *S. pombe* (Zhang et al., 2008, 2013; Baumer et al., 2009; Campion et al., 2010; Boulisfane et al., 2011; Lotti et al., 2012; Praveen et al., 2012; Garcia et al., 2013, 2016; Tisdale et al., 2013; Maeda et al., 2014; See et al., 2014; Sleigh et al., 2014; Miller et al., 2016; Bernabo et al., 2017; Jangi et al., 2017; O’Hern et al., 2017). Though multiple genes and pathways have been described, common molecular changes shared by these SMA models, except for snRNP formation, remain to be uncovered.

Mitochondrial dysfunctions have been described in human iPSC and zebrafish models of SMA (Xu C.C. et al., 2016; Boyd et al., 2017). Defective mitochondrial gene expression and mitochondrial dysfunction were detected in pre-symptomatic motor neurons of a mouse SMA model (Miller et al., 2016). Similarly, defective mitochondrial biogenesis was found in the muscles of SMA patients (Ripolone et al., 2015). We found that *smn-1(ok355)* caused reduced expression of multiple mitochondrial genes in *C. elegans*.

The molecular mechanisms underlying the effects of *SMN* on mitochondrial functions and gene expression remain to be understood. A recent study in *C. elegans* found that SMN-1 promotes the localization of mitochondria to the I bands of body wall muscles, which requires the Arp2/3 actin-nucleation complex (Schultz et al., 2017). Consistently, *smn-1* deficiency causes defects in mitochondrial morphology and subcellular position in body wall muscles, a function likely independent of the regulation of RNA splicing by SMN-1 (Schultz et al., 2017). Together, these findings suggest that SMN can impact mitochondrial functions by different mechanisms, including the regulation of mitochondrial gene expression, mitochondrial morphology and subcellular localization.

Defects in the morphology, number and endocytosis of synaptic vesicles were described in human (Hosseinibarkooie et al., 2016; Riessland et al., 2017), mouse (Kong et al., 2009; Torres-Benito et al., 2011; Tejero et al., 2016; Xu Y. et al., 2016) and *C. elegans* (Dimitriadi et al., 2016) models of SMA. V-ATPases are proton pumps that regulate the acidity of a variety of cellular organelles, including lysosomes, exosomes, endosomes and synaptic vesicles (Futai, 2007). The reduced expression of multiple V-ATPase genes in *smn-1(ok355)* mutants might compromise the luminal acidity of these vesicles, causing defects in endosome, lysosome and/or synaptic vesicles. Hence, it is plausible that reduced V-ATPase expression could contribute to the vesicle defects in *SMN* mutants.

Multiple genes related to lifespan, motor functions and neuronal activities were also affected in *smn-1(ok355)* mutants (Supplementary Table S7). The related KEGG pathways or GO terms, however, were not significantly affected based on bioinformatics analyses (Supplementary Table S14). Though the expression of these genes is yet to be validated, it is interesting to note that the GO terms containing the largest numbers of DEGs include determination of adult lifespan, locomotion and neurogenesis (Supplementary Table S14). Further analyses of these genes might facilitate the understanding of the deterioration of the motor behavior and shortened lifespan of *smn-1(ok355)* mutants.

### Up-Regulated Expression of Histone Genes in *SMN* Mutants

In mammals, SMN is required for the formation of U7 snRNP (Pillai et al., 2003) that cleaves after the stem-loop structure of replication-dependent histone transcripts to generate *poly(A)-* transcripts (Hoffmann and Birnstiel, 1990). In mouse cells, *SMN* deficiency leads to defective U7 snRNP formation and increased levels of *poly(A)+* transcripts of replication-dependent histone genes (Tisdale et al., 2013). A previous transcriptome study on the spinal cord of a mouse model of SMA also found up-regulation of the histone cluster 1 genes (Baumer et al., 2009), which are replication-dependent. We found that *poly(A)+* transcripts of replication-dependent histone genes were similarly up-regulated in *smn-1(ok355)* mutants. However, this up-regulation might not involve defective U7 snRNP formation like that in mouse, as conserved U7 snRNA sequence was not identified in the *C. elegans* genome by bioinformatics analyses (Dávila López and Samuelsson, 2008). Interestingly, *C. elegans* appears to have evolved an alternative pathway to cleave the transcripts of replication-dependent histone genes using the CSR-1 endogenous RNAi pathway in germline (Avgousti et al., 2012). It remains to be determined whether the CSR-1 RNAi pathway extends its effect to neurons and muscles.

Besides *poly(A)+* transcripts, the total transcripts of three out of four replication-dependent histone genes, as well as four replication-independent histone genes, were also up-regulated in *smn-1(ok355)* mutants (Figure 3F). This result implies that *smn-1* might broadly regulate histone gene expression by affecting their transcription, stability and/or degradation, the detailed mechanism of which remains to be elucidated.

Considering the functional multiplicity of SMN on RNA splicing, transcription, translation, mRNA trafficking and ncRNA biogenesis, etc. (Singh et al., 2017), caution has to be exercised when interpreting the expression changes of the aforementioned genes as SMN-1-specific. For example, some or all the changes of gene expression might be similarly caused by defects in transcription, splicing or RNA metabolism involving other biomolecules. Therefore, global gene expression studies on mutants of more splicing factors or gene expression regulators are warranted to clarify these possibilities.

### The Effect of *smn-1* on RNA Splicing and Gene Expression

Studies in human (Jangi et al., 2017), mouse (Zhang et al., 2008, 2013; Van Alstyne et al., 2018), *Drosophila* (Garcia et al., 2016), and *S. pombe* (Campion et al., 2010) suggest that SMN deficiency can cause intron retention and/or exon skipping, both likely due to reduced recognition of 3′ splice sites.

By examining nine previously validated alternative splicing events (Zhou et al., 2018), we found that *smn-1(ok355)* caused reduced recognition of weak 3′ splice sites in two genes (Figure 5A,C). This finding is consistent with our previous studies using the *tos-1* reporter transgenes (Ma et al., 2011), in which we found that weak 3′ splice sites were preferably affected in *smn-1(ok355)* mutants (Gao et al., 2014).

The molecular mechanism underlying the down-regulated expression of genes in *smn-1(ok355)* mutants remains to be elucidated. It is plausible that *smn-1* deficiency causes defective splicing of these transcripts and as a result generates pre-mature stop codons. Such transcripts are often degraded by non-sense-mediated mRNA decay (NMD) (He and Jacobson, 2015), causing down-regulated expression of the target genes. For the same reason, these defectively spliced transcripts will be undetectable by RT-PCR, which might explain why we were not able to detect obviously altered splicing in 18 down-regulated DEGs (Supplementary Table S13). Interestingly, similar observation was made with a mouse model of SMA, in which Baumer et al. (2009) found that altered splicing events were very few in the spinal cord at early or pre-symptomatic stages but abundant at a late symptomatic stage. Apparently, NMD could similarly underlie the shortage of detectable splicing events in this mouse model. We postulate that, besides NMD, other mechanisms might also be involved in the down-regulation. For example, *smn-1* deficiency might cause reduced transcription or transcript stability of these genes. Future studies in NMD mutant backgrounds (Mango, 2001) should provide new insights into these potential mechanisms.

In *C. elegans*, knocking down *smn-1* specifically in motor neurons can cause degeneration (Gallotta et al., 2016), suggesting that *smn-1*, like *SMN1* in human, is essential for the survival of motor neurons. In both species, however, the molecular changes underlying the motor neuron degeneration have been elusive. Similar to the findings in this study, our previous analysis of the spinal cord motor neurons of a mouse SMA model also identified defective expression of numerous mitochondrial genes and defects in mitochondrial bioenergetics (Miller et al., 2016). It is plausible that defective mitochondrial gene expression, even at a partial level, would harm the health of spinal cord neurons. However, how much this defect would contribute to the degeneration remains to be understood. Since many other genes are also affected by *smn-1*, a key future task is to determine whether and how these genes affect the survival and functions of motor neurons in *C. elegans*.

Our validation of the down-regulated DEGs (Figure 3A–D) indicates that the expression of some genes in *smn-1(ok355)* mutants was reduced to ∼50% of the wild-type levels. Though not dramatic, this level of change appears reasonable, as it was detected at the pre-symptomatic stage of *smn-1(ok355)* mutants when a residual amount of maternal *smn-1* product was probably still expressed. The defects in gene expression might become more severe when the mutants enter later developmental stages, in which the maternal *smn-1* will be completely depleted. The expression of the *plst-1* gene provides such an example (see below). In addition, if any of these DEGs contribute to the defects of *smn-1(ok355)* mutants, it is more likely that they do so together with other DEGs, while the effects of an individual or a few genes could be weak. Hence, it is important to identify a comprehensive list of DEGs before we can have a more impartial understanding of the molecular changes related to the defects of *smn-1(ok355)* mutants.

The human *PLS3* gene was identified as a protective modifier of SMA (Oprea et al., 2008). We previously found that the expression of *plst-1*, the *C. elegans* ortholog of *PLS3*, was significantly down-regulated in *smn-1(ok355)* mutants at later developmental stages (3 days or 5 days after the L1 stage) (Gao et al., 2014). In the RNA-Seq results, *plst-1* was not identified as a DEG (fold change = 0.797, *p*-value = 0.77), suggesting that *plst-1* expression might not be severely affected at the pre-symptomatic stage of *smn-1(ok355)* mutants.

The expression levels of U snRNAs could not be extracted from the RNA-Seq results because only *poly(A)+* mRNAs were examined by the RNA-Seq experiments (see section “Materials and Methods”). We therefore used RT-qPCR to quantify their expression. The results showed that U2 and U4 were significantly up-regulated in *smn-1(ok355)* mutants (Supplementary Figure S5). In previous studies, we found that U2, U4, and U6 were also up-regulated in *smn-1(ok355)* mutants at later developmental stages (Gao et al., 2014). Though it is unclear what mechanism underlies this change, one possibility is that the up-regulation of these U snRNAs is a feedback response to *smn-1* deficiency. Alternatively, *smn-1* might negatively affect the expression of U2, U4, and U6. It remains to be determined whether the formation of U snRNPs is also affected in *smn-1(ok355)* mutants at the pre-symptomatic stage. Nevertheless, these results suggest that *smn-1* can exert differential effects on the expression of U snRNAs and uncovering the underlying mechanism might facilitate our understanding of *SMN* functions.

### *smn-1* and *uaf-1* Interact to Affect Alternative Splicing and Gene Expression

Intriguingly, *smn-1* was required for the recognition of a cryptic 5′ splice site in *uaf-1(n4588)* background (Figure 5D, *zipt-2.4*) (Zhou et al., 2018). Such an activity of *smn-1* is comparable to that of the *rbm-5* gene (Zhou et al., 2018), which encodes an RNA-binding motif-containing splicing factor homologous to the tumor suppressor RBM5 (Sutherland et al., 2010). We postulate that RBM-5 might facilitate the recognition of this cryptic 5′ splice site at the initiation of splicing with direct or indirect assistance from the UAF-1(n4588) mutant protein, while snRNPs assembled by SMN-1 are required in parallel or in subsequent splicing reactions. In the absence of either RBM-5 or SMN-1, splicing at this cryptic site would not proceed, leading to the suppression. This finding raised the possibility that regulated snRNP formation might be utilized to increase the fidelity of RNA splicing by limiting splicing at cryptic sites.

We hypothesize two potential mechanisms that underlie the suppression of *smn-1(ok355)* by *uaf-1(n4588)* (Gao et al., 2014). Under one circumstance, the altered splicing or expression of some genes caused by *smn-1(ok355)* might be ameliorated by *uaf-1(n4588)*, which would lead to the suppression. Under an alternative circumstance, the altered splicing or expression of a new set of genes might impact the phenotype of *smn-1(ok355); uaf-1(n4588)* double mutants, leading to the suppression.

It appears that the splicing of the *abt-5* gene (Figure 5A) is consistent with the first circumstance: the presence of *uaf-1(n4588)* suppressed the defective splicing at the 3′ splice site in *smn-1(ok355)* mutants. We found that *uaf-1(n4588)* also suppressed the up-regulated expression of two genes in *smn-1(ok355)* background (Supplementary Figure S3B). Considering that *uaf-1(n4588)* can increase or decrease the recognition of 3′ splice sites in a gene-specific manner (Ma and Horvitz, 2009; Ma et al., 2011; Zhou et al., 2018), such a suppression might result from reduced splicing of these two genes caused by *uaf-1(n4588)*, which might generate splicing isoforms that are degraded by NMD. Alternatively, *uaf-1(n4588)* might affect a gene that is required for the normal expression of these genes. Similarly, expression studies in an NMD mutant background should help dissect these potential mechanisms.

In short, we identified the defective expression of a broad spectrum of genes that encode mitochondrial, V-ATPase, histone, UGT and cytochrome P450 proteins as a probable pre-symptomatic effect of *smn-1* deficiency in *C. elegans*. The splicing of a subset of genes was also partially affected at this stage. Though the molecular mechanisms underlying this phenomenon remain to be investigated, our results, together with findings in other SMA models, suggest a cross-species effect of SMN on mitochondrial and histone gene expression. Future examination of the expression of more genes and analyses of their functions should further the understanding of SMN functions.

## Materials and Methods

### Strains

*Caenorhabditis elegans* strains were grown at 20°C, unless otherwise indicated. N2 (Bristol) was the reference wild-type strain. Strains used in this study include:

LM99 *smn-1(ok355) I/hT2[bli-4(e937) let-?(q782) qIs48](I, III)* (Briese et al., 2009)

MT16492 *uaf-1(n4588) III* (Ma and Horvitz, 2009)

CSM1111 *smn-1(ok355) I/hT2[bli-4(e937) let-?(q782) qIs48] (I; III); uaf-1(n4588) III*

CSM1112 *smn-1(ok355) I/hT2[bli-4(e937) let-?(q782) qIs48] (I; III); macEx588[hil-3::mCherry; myo-3p::GFP]*

CSM1113 *smn-1(ok355) I/hT2[bli-4(e937) let-?(q782) qIs48] (I; III); macEx589[his-35::mCherry; myo-3p::GFP]*

CSM1114 *smn-1(ok355) I/hT2[bli-4(e937) let-?(q782) qIs48] (I; III); macEx590[his-41::mCherry; myo-3p::GFP]*

CSM1111 was grown at 15°C before being analyzed at 20°C.

### RNA-Seq and Transcriptome Analyses

*smn-1(ok355)* homozygous mutants arrest at late larval stages and were maintained as heterozygotes using the balancer *hT2 (I; III)*. Heterozygous mutants or wild-type animals were synchronized by bleaching and allowed to grow for 2 days post the L1 larval stage. For *smn-1(ok355)* single mutants or *smn-1(ok355); uaf-1(n4588)* double mutants, ∼2,500 individuals were picked for each biological replicate under a fluorescence dissecting microscope (Olympus SZX16). Animals were washed with M9 three times and kept in M9 for 3 h to remove intestinal bacteria. Total RNAs were extracted using TRI Reagent Solution according to manufacturer’s instructions (Invitrogen).

RNA-Seq was performed by Annoroad Gene Technology (Beijing). Specifically, mRNAs were enriched with oligo(dT) beads. The constructed libraries were sequenced as 150 bp paired-ends on an Illumina platform. Raw data was filtered by Perl scripts using the following criteria: (1) reads containing more than five adapter-polluted bases were filtered out; (2) reads with the number of low quality bases (Phred quality value < 19) in more than 15% of total bases were removed; (3) reads with the number of N bases accounting for more than 5% were removed; (4) both reads would be filtered out if either one of the paired-end reads was adaptor-polluted.

More than 39 million mapped reads and more than 6 GB of clean bases were obtained for each sample (Supplementary Table S15). Bowtie/Bowtie2 was used for building *C. elegans* genome index, and clean data was mapped to the reference genome (WS235) using TopHat v2.0.12. Differential gene expression analysis was performed by DESeq v1.14.0. The *p*-value was adjusted by Benjamini and Hochberg’s method as *q*-value for controlling false discovery rate (FDR). Genes with *q* ≤ 0.05 and | log_2__ratio|≥ 1 were identified as DEGs.

Alternative splicing events were predicted by ASprofile v1.0.4^4^, a package of software programs for analyzing alternative splicing events from RNA-Seq data. The ratio of a given alternative splicing event was determined by the following formula, e.g., the percentage of an intron retention event = FPKM_withintron_/(FPKM_withintron_ + FPKM_withoutintron_). The ratios of the same splicing event were compared between genotypes to identify potential splicing differences.

KEGG and GO enrichment analyses on the DEGs were implemented by hypergeometric test using all genes in the *C. elegans* genome (WS235) with annotated functions in the two databases as the background gene set. *P*-value for the enrichment level was calculated and adjusted as *q*-value. KEGG or GO terms with *q* ≤ 0.05 were considered significantly affected by the DEGs.

In total, two biological replicates for wild-type animals or *smn-1(ok355); uaf-1(n4588)* double mutants and three biological replicates for *uaf-1(n4588)* or *smn-1(ok355)* single mutants were analyzed by RNA-Seq. We examined the correlation coefficients between the biological replicates for each genotype (Supplementary Figure S6) and found that except for *smn-1(ok355); uaf-1(n4588)*, replicates of all other genotypes were highly correlated (*r* > 0.9). The coefficient for the two replicates of *smn-1(ok355); uaf-1(n4588)* mutants was 0.8260 (Supplementary Figure S6), which might explain why we detected a significant number of false negative DEGs in these mutants (Supplementary Figure S3).

### RT-PCR Experiments

Synchronized animals were grown on NGM plates for 2 days post L1. For *smn-1(ok355)* and *smn-1(ok355); uaf-1(n4588)* mutants, ∼250 animals were picked for each biological replicate. Total RNAs were prepared using TRI Reagent Solution (Invitrogen) with 15 μg yeast tRNA (Invitrogen) added as co-precipitant, followed by RNase-Free DNase I (TAKARA) digestion. cDNAs were synthesized with random hexamers using Maxima First Strand cDNA Synthesis Kit (Thermo Scientific) or with oligo(dT)_20_ primers (only for replication-dependent histone genes) using SuperScript IV First-Strand Synthesis System (Invitrogen). 2 μg total RNA (including yeast tRNA as co-precipitant) from each sample was used for RT experiments. qPCR was performed using Maxima SYBR Green qPCR Master Mix (Thermo Scientific) on a Bio-Rad CFX96 real-time cycler. The reaction mix contains 2 μL of diluted template (derived from 5 ng total RNA) and 0.3 μM of each primer in a final volume of 15 μL. Thermal cycling was performed using a two-step cycling protocol: 10 min at 95°C, followed by 40 cycles of 15 s at 95°C and 60 s at 60°C. Bio-Rad CFX Manager v3.0 was used to calculate the relative expression level of each gene with *tba-1* as the reference gene. Melting curve for each sample contained one single peak. Alternative splicing was detected using the following PCR protocol: 90 s at 94°C, followed by 40 cycles of 15 s at 94°C, 15 s at 60°C and 30 s at 72°C, with a final extension of 3 min at 72°C. The proportion of each splice isoform was quantified using ImageJ 1.52a. Three biological replicates were analyzed for each genotype. PCR primers are listed in Supplementary Table S16.

### Plasmids

*his-41* expression plasmid was constructed using the In-Fusion HD Cloning Kit (Clontech) following the manufacturer’s instruction. First, the genomic fragment containing the full-length *his-41* CDS and a 1.1 kb endogenous promoter was amplified and subcloned into pMD18T vector (Takara). Second, the *mCherry* gDNA from the pCFJ90 (*myo-2p::mCherry*) plasmid (Frokjaer-Jensen et al., 2008) and a 3′ UTR fragment (0.9 kb downstream of the *his-41* stop codon) were amplified and subcloned into pMD18T-*his-41p::his-41* gDNA backbone using the *Kpn*I site.

The *his-35* and *hil-3* expression plasmids were constructed in a similar manner. For *his-35*, a 1.3 kb promoter and a 1.2 kb 3′ UTR were used. For *hil-3*, a 1.8 kb promoter and a 0.6 kb 3′ UTR were used. All plasmids were verified by sequencing. Primers for plasmid construction are listed in Supplementary Table S16.

### Transgene Experiments

Germline transgene experiments were performed as described (Mello et al., 1991). The transgenic mixture contained 20 ng/μL of the transgene of interest with 20 ng/μL of pPD95_86-*myo-3p::GFP* as co-injection marker.

### *C. elegans* Microscopy

Synchronized animals were mounted on 2% (vol/vol) agar pads on day 2 post the L1 larval stage and immobilized using 40 mM NaN_3_ in M9 buffer. Images were captured with Zeiss 880 confocal microscope (10× objective). Total fluorescence intensity was quantified as un-calibrated OD, and fluorescence density was calculated using total intensity divided by area using ImageJ 1.52a.

### Statistics

*P*-values were determined by two-tailed unpaired Student’s *t-*test for pairwise comparison or Bonferroni multiple comparison with one-way ANOVA for multiple comparison using GraphPad Prism 7.0 software.

## Author Contributions

XG and LM designed the experiments. XG performed the genetic, molecular, and transcriptome analyses with the assistance of JX, HC, DX, WP, and CZ. XG, YCM, and LM wrote the manuscript. LM managed the project.

## Conflict of Interest Statement

The authors declare that the research was conducted in the absence of any commercial or financial relationships that could be construed as a potential conflict of interest.

## Footnotes

1 https://www.genome.jp/kegg/

2 www.wormbase.org

3 http://www.geneontology.org

4 https://ccb.jhu.edu/software/ASprofile/

## Supplementary Material

FIGURE S1 Expression levels of predicted DEGs not confirmed by RT-qPCR. **(A)** Mitochondrial genes. Genes in red are encoded by the mitochondrial genome and genes in black by the nuclear genome. **(B)** V-ATPase genes. **(C)** UGT genes. **(D)** CYP genes. Each dataset is the average of three biological replicates. Statistics: two-tailed unpaired Student’s *t*-test. Error bars: standard errors.

FIGURE S2 Comparison of affected KEGG pathways and GO cellular component terms between *smn-1(ok355)* single and *smn-1(ok355); uaf-1(n4588)* double mutants. **(A)** Comparison of affected KEGG pathways between *smn-1(ok355)* single and *smn-1(ok355); uaf-1(n4588)* double mutants using wild type as reference. **(B)** Comparison of affected GO cellular component terms between *smn-1(ok355)* single and *smn-1(ok355); uaf-1(n4588)* double mutants. Note, the KEGG pathways and GO terms for *smn-1(ok355)* single mutants, which are presented in Figure 2, are shown here for comparison purpose. The colors of the scale bars indicate the statistical significance (*q*-value) of the affected pathways or terms, with *q*-value above 0.05 considered non-significant.

FIGURE S3 RT-qPCR quantification of mitochondrial and V-ATPase gene expression in *smn-1(ok355); uaf-1(n4588)* double mutants. **(A)** List of selected genes with predicted significant changes in *smn-1(ok355)* single mutants but insignificant changes in *smn-1(ok355); uaf-1(n4588)* double mutants. Fold changes and *q* values are derived from RNA-Seq results. **(B)** Expression levels of mitochondrial genes. **(C)** Expression levels of V-ATPase genes. Each dataset is the average of three biological replicates. Statistics: Bonferroni multiple comparison with one-way ANOVA. ^∗^*p* < 0.05; ^∗∗^*p* < 0.01; ^∗∗∗^*p* < 0.001. Error bars: standard errors.

FIGURE S4 *smn-1(ok355)* does not obviously affect the alternative splicing of five other genes in the wild-type or *uaf-1(n4588)* background. Gene names are on the left. Representative gel pictures, gene structures and splicing quantifications are shown from left to right, with genotypes indicated at the bottom. Red arrows: positions of PCR primers. Each dataset is the average of three biological replicates. Statistics: Bonferroni multiple comparison with one-way ANOVA. ^∗^*p* < 0.05; ^∗∗^*p* < 0.01; ^∗∗∗^*p* < 0.001. Error bars: standard errors.

FIGURE S5 RT-qPCR quantification of U snRNA expression in *smn-1(ok355)* mutants at the pre-symptomatic stage. cDNAs of U snRNAs were generated by reverse transcription using random oligo primers. Each dataset is the average of three biological replicates. Statistics: two-tailed unpaired Student’s *t*-test. ^∗^*p* < 0.05, ^∗∗∗^*p* < 0.001. Error bars: standard errors.

FIGURE S6 Scatter plots comparing the expression levels of all detectable genes between biological replicates of different genotypes. **(A)** N2a sample vs. N2b sample comparison. **(B)** *smn-1(ok355)*a vs. *smn-1(ok355)*b. **(C)** *smn-1(ok355)*a vs. *smn-1(ok355)*c. **(D)** *smn-1(ok355)*b vs. *smn-1(ok355)*c. **(E)** *uaf-1(n4588)*a vs. *uaf-1(n4588)*b. **(F)** *uaf-1(n4588)*a vs. *uaf-1(n4588)*c. **(G)** *uaf-1(n4588)*b vs. *uaf-1(n4588)*c. **(H)** *smn-1(ok355); uaf-1(n4588)*a vs. *smn-1(ok355); uaf-1(n4588)*b. The correlation coefficient (*r*) for each comparison is indicated. Each dot represents a unique gene.

TABLE S1 List of all DEGs in *smn-1(ok355)* single mutants. Genes were sorted to an up-regulated group and a down-regulated group. DEGs with gradually increasing *q*-values are listed from the top to the bottom.

TABLE S2 List of top 200 up-regulated DEGs and top 200 down-regulated DEGs in *smn-1(ok355)* single mutants. DEGs with gradually decreasing fold changes are listed from the top to the bottom. Up-regulated DEGs are highlighted in red, and down-regulated DEGs are highlighted in green.

TABLE S3 RNA-Seq raw sequencing reads and FPKMs of *smn-1* transcripts in different biological replicates.

TABLE S4 List of DEGs in affected KEGG pathways in *smn-1(ok355)* single mutants.

TABLE S5 List of non-redundant DEGs in affected KEGG pathways in *smn-1(ok355)* single mutants. Shared DEGs between *smn-1(ok355)* single mutants and *smn-1(ok355); uaf-1(n4588)* double mutants are highlighted in red.

TABLE S6 List of non-redundant DEGs in affected GO cellular component terms in *smn-1(ok355)* single mutants. Shared DEGs between *smn-1(ok355)* single mutants and *smn-1(ok355); uaf-1(n4588)* double mutants are highlighted in red. Some genes annotated in GO but not in KEGG, e.g., *dhs-26* and *T22B7.7*, were validated in Supplementary Figure S3.

TABLE S7 List of DEGs in GO terms related to lifespan, motor functions or neuron activities in *smn-1(ok355)* mutants. DEGs related to mitochondrion are highlighted in red, and those related to V-ATPases are highlighted in green. *atp-2*, *C47E12.2*, *msra-1* and *W03A5.1* are related to both lifespan and motor functions.

TABLE S8 List of DEGs encoding mitochondrial proteins, V-ATPases, UDP glycosyltransferases, cytochrome P450 proteins and histones in *smn-1(ok355)* single mutants. Genes selected for RT-qPCR validation are highlighted in red. Validation results are indicated in Column B.

TABLE S9 List of DEGs in affected KEGG pathways in *smn-1(ok355); uaf-1(n4588)* double mutants.

TABLE S10 Affected GO terms in *uaf-1(n4588)* single mutants.

TABLE S11 List of DEGs in GO terms from the comparison between *smn-1(ok355); uaf-1(n4588)* and *smn-1(ok355)*. DEGs related to mitochondrion are highlighted in red. *vit-1* to *vit-6* are related to both extracellular region and nutrient reservoir activity. *F35D2.3*, *slt-1* and *W03F11.4* are related to both extracellular region and motor functions or neuron activities.

TABLE S12 List of DEGs the expression of which was significantly changed to the opposite direction in *smn-1(ok355); uaf-1(n4588)* vs. *smn-1(ok355)* comparison relative to the *smn-1(ok355)* vs. N2 comparison.

TABLE S13 List of mitochondrial and V-ATPase DEGs with altered splicing in *smn-1(ok355)* mutants as predicted by RNA-Seq.

TABLE S14 DEG-associated GO terms (biological processes) related to lifespan, motor functions or neuron activities in *smn-1(ok355)* mutants.

TABLE S15 Total RNA-Seq reads of each biological replicate.

TABLE S16 List of primers for RT-PCR experiments and transgene construction.
