# Supplementary figures and images for: Defective Expression of Mitochondrial, Vacuolar H+-ATPase and Histone Genes in a C. elegans Model of SMA
Source: Front Genet. 2019 May 3;10:410. doi: 10.3389/fgene.2019.00410 (PMC6509145; doi:10.3389/fgene.2019.00410)

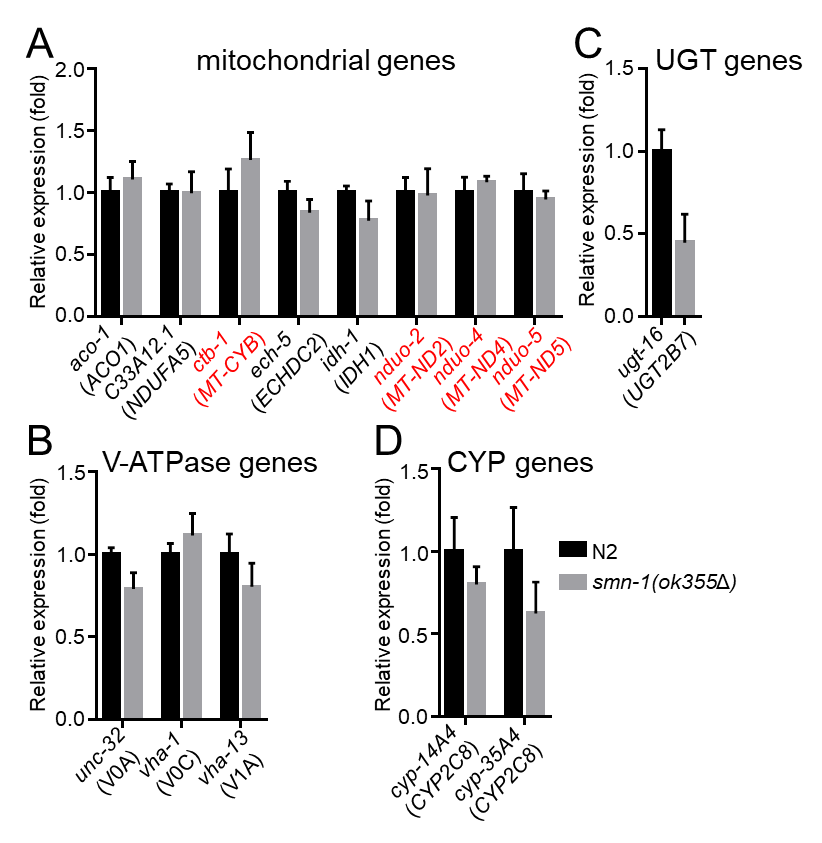

Supplement: FIGURE S1 — Expression levels of predicted DEGs not confirmed by RT-qPCR. (A) Mitochondrial genes. Genes in red are encoded by the mitochondrial genome and genes in black by the nuclear genome. (B) V-ATPase genes. (C) UGT genes. (D) CYP genes. Each dataset is the average of three biological replicates. Statistics: two-tailed unpaired Student’s t-test. Error bars: standard errors. [file Data_Sheet_1.zip › Figure S1.tif]

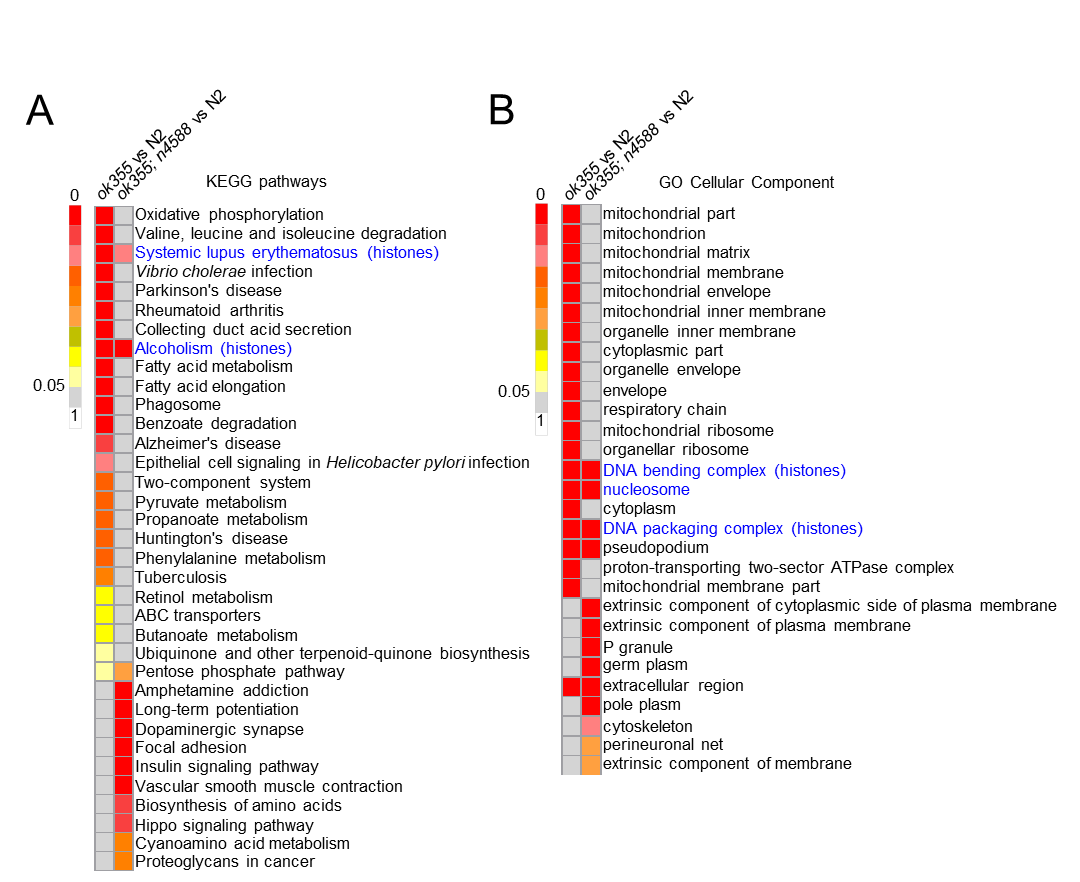

Supplement: FIGURE S1 — Expression levels of predicted DEGs not confirmed by RT-qPCR. (A) Mitochondrial genes. Genes in red are encoded by the mitochondrial genome and genes in black by the nuclear genome. (B) V-ATPase genes. (C) UGT genes. (D) CYP genes. Each dataset is the average of three biological replicates. Statistics: two-tailed unpaired Student’s t-test. Error bars: standard errors. [file Data_Sheet_1.zip › Figure S2.tif]

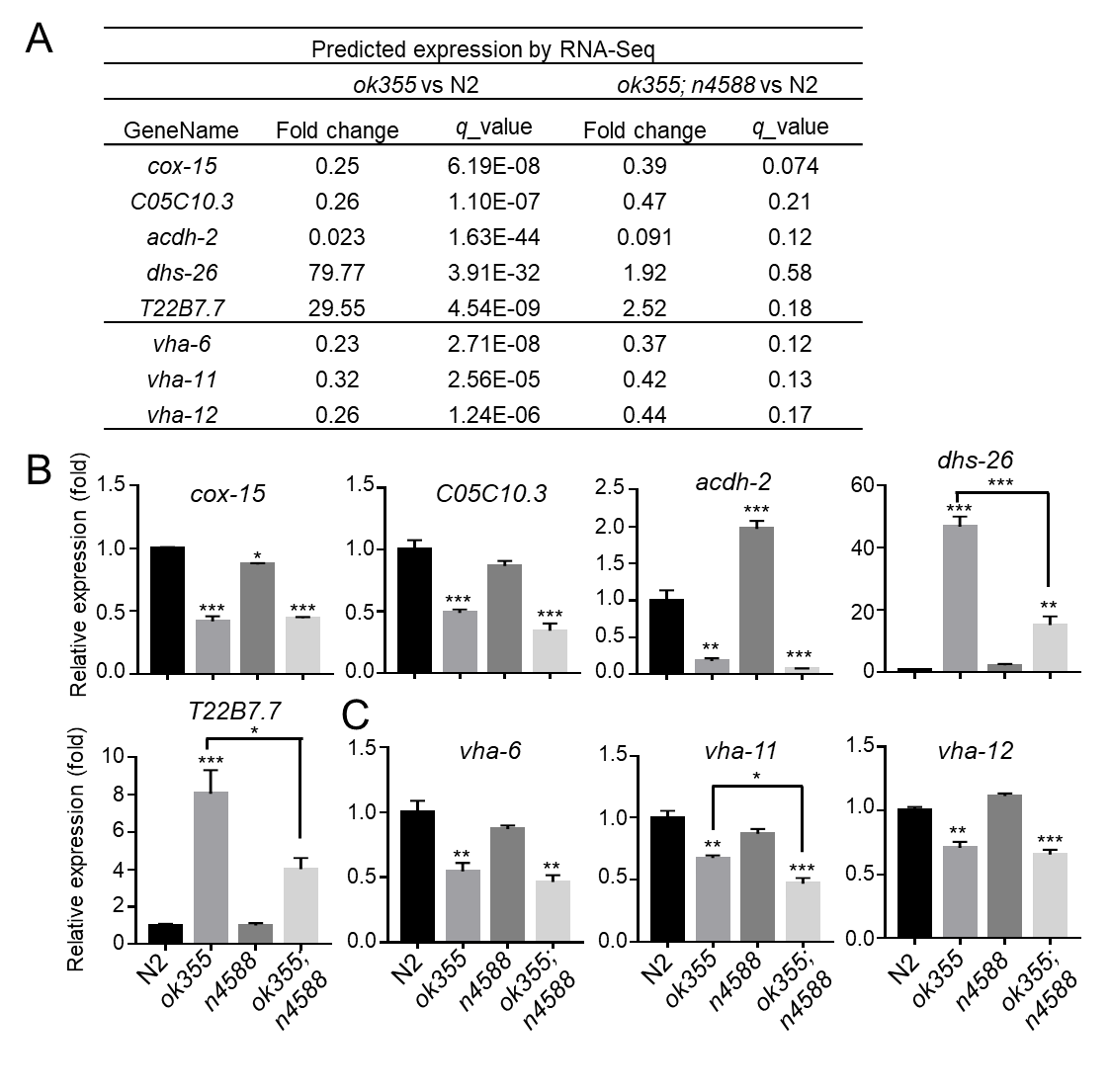

Supplement: FIGURE S1 — Expression levels of predicted DEGs not confirmed by RT-qPCR. (A) Mitochondrial genes. Genes in red are encoded by the mitochondrial genome and genes in black by the nuclear genome. (B) V-ATPase genes. (C) UGT genes. (D) CYP genes. Each dataset is the average of three biological replicates. Statistics: two-tailed unpaired Student’s t-test. Error bars: standard errors. [file Data_Sheet_1.zip › Figure S3.tif]

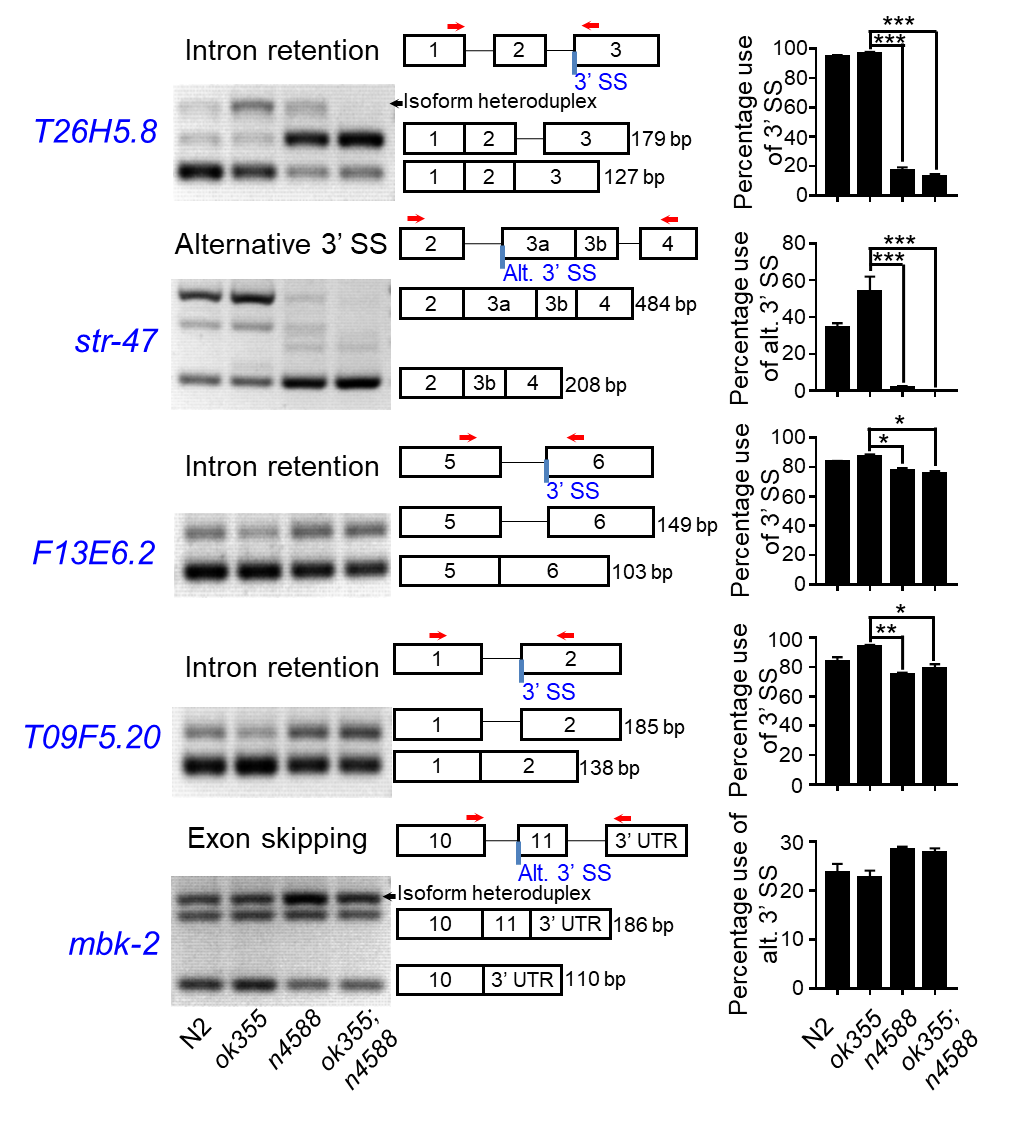

Supplement: FIGURE S1 — Expression levels of predicted DEGs not confirmed by RT-qPCR. (A) Mitochondrial genes. Genes in red are encoded by the mitochondrial genome and genes in black by the nuclear genome. (B) V-ATPase genes. (C) UGT genes. (D) CYP genes. Each dataset is the average of three biological replicates. Statistics: two-tailed unpaired Student’s t-test. Error bars: standard errors. [file Data_Sheet_1.zip › Figure S4.tif]

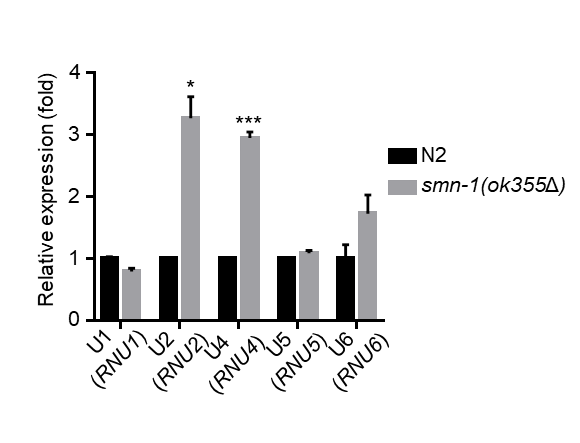

Supplement: FIGURE S1 — Expression levels of predicted DEGs not confirmed by RT-qPCR. (A) Mitochondrial genes. Genes in red are encoded by the mitochondrial genome and genes in black by the nuclear genome. (B) V-ATPase genes. (C) UGT genes. (D) CYP genes. Each dataset is the average of three biological replicates. Statistics: two-tailed unpaired Student’s t-test. Error bars: standard errors. [file Data_Sheet_1.zip › Figure S5.tif]

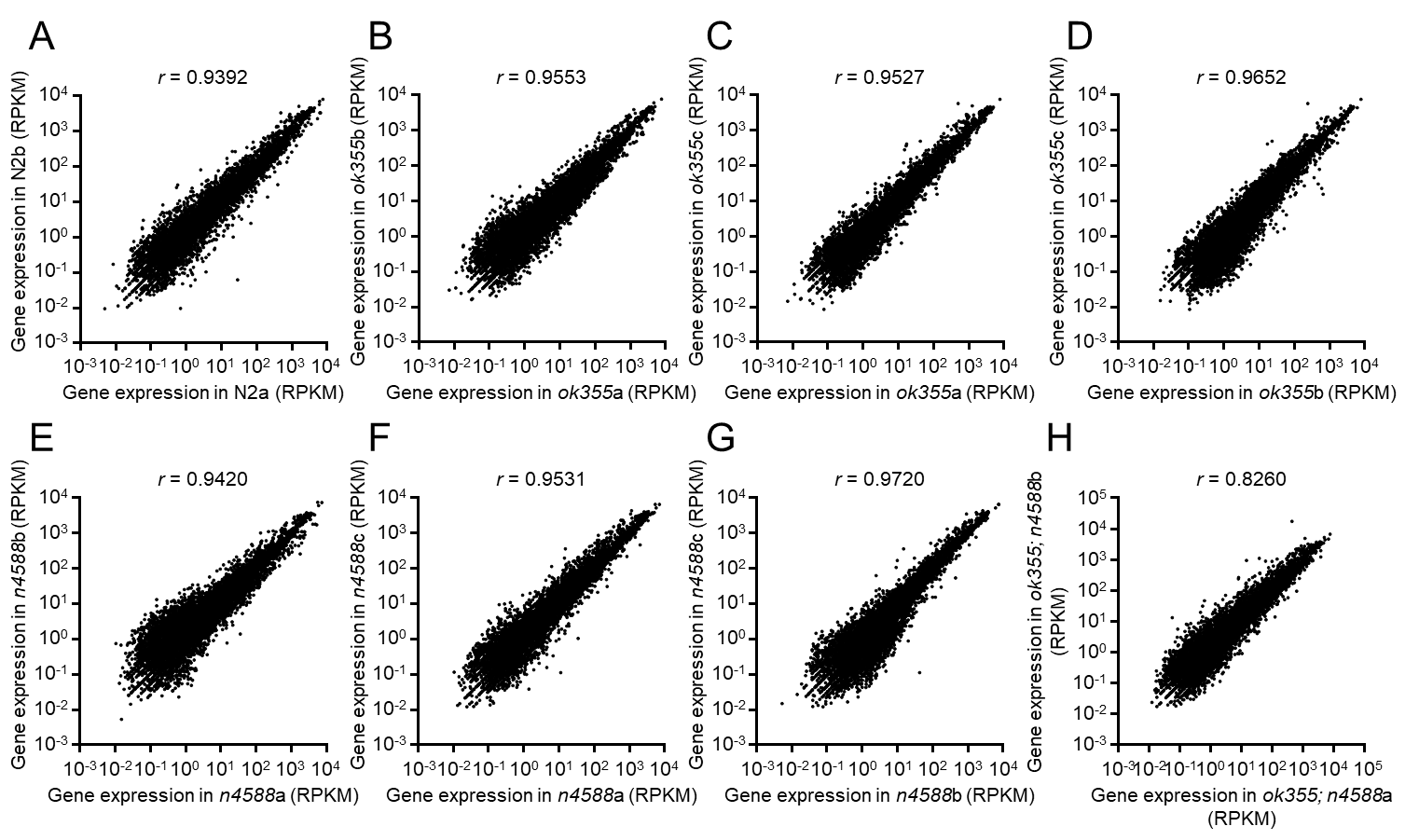

Supplement: FIGURE S1 — Expression levels of predicted DEGs not confirmed by RT-qPCR. (A) Mitochondrial genes. Genes in red are encoded by the mitochondrial genome and genes in black by the nuclear genome. (B) V-ATPase genes. (C) UGT genes. (D) CYP genes. Each dataset is the average of three biological replicates. Statistics: two-tailed unpaired Student’s t-test. Error bars: standard errors. [file Data_Sheet_1.zip › Figure S6.tif]
